# A contrastive analysis of laser heating between the human and guinea pig cochlea by numerical simulations

**DOI:** 10.1186/s12938-016-0190-1

**Published:** 2016-05-23

**Authors:** Kaiyin Zhang, Yulong Zhang, Ji Li, Qiuling Wang

**Affiliations:** School of Physics and Electronics, Fuyang Normal College, Fuyang, 236037 Anhui China; School of Mathematic and Physics, Jiangsu University of Science and Technology, Zhenjiang, 212003 Jiangsu China

**Keywords:** Laser stimulation, Cochlea, Guinea pig, Human, Laser safety

## Abstract

**Background:**

The photo-thermal effect has been hypothesised to be one of the most possible biophysical mechanisms for laser-cochlea stimulation. However, there is a lack of studies to date for direct assessing laser heating in humans due to the large body of evidence required to demonstrate safety and efficacy. Instead, the majority focus on animals like the guinea pig, from which a number of valuable results have been gained. However, in light of the increasing need to improve laser safety, it has became necessary to find out whether studies on animals can shed light on safe laser parameters in the human cochlea. Hence, we conducted this contrastive analysis of laser heating between the human and guinea pig cochlea with the aim of assisting further investigations in this field.

**Methods:**

In this work, a 3D symmetrical model was adopted to simplify the spiraled cochlea. With attention focused on the effect of heat conduction, the time-dependent heat equation was solved using finite element method with the COMSOL Script. In the simulations, cochleae with different sizes and various boundary thermal conditions were utilized.

**Results:**

Laser heating in both cochleae has a similar trend. In the first stage, or at the beginning of the laser heating, both cochleae increased their temperatures rapidly. In the second stage in which the laser heating reached a quasi-steady stage, the peak temperatures began to rise slowly as more laser pulses were applied. However, three differences of the laser heating were observed. The first is regarding the temperature rise. The results show that laser heating in guinea pig is higher than that in human under the same laser parameters. The second difference is the fluctuation of temperature rise at the center of the modiolus. There is a larger fluctuation of temperature rise in the guinea pig cochlea, compared with that in the human cochlea. The third one is the time for reaching a steady thermal state. The results show that the guinea pig cochlea takes longer time to reach a steady thermal state than the human cochlea. Those differences are mainly attributed to the distinctive thermal boundaries and the various sizes of the two cochleae.

**Conclusions:**

This study finds that the laser heating in the guinea pig cochlea is higher than that in the human cochlea under the condition of the same laser parameters. However, laser stimulation still displays a high spatial selectivity in both cochleae despite the effects of heat conduction. The results indicate that experimental studies on the guinea pig could appropriately be an alternative model for the sake of laser safety.

## Background

In recent years, laser light with a wide range of wavelengths has been used successfully to stimulate auditory responses of gerbils, mice, guinea pigs and cats [1–5]. Besides experiments in animals, Fishman et al. [6] conducted a pioneering study of optical stimulation of the auditory nerve in a patient who required of surgical removal of a large meningioma. Although many questions in research remain to be answered in terms of laser stimulation, the initial results are promising beginnings. Among various biophysical mechanisms for the laser stimulation, three types of hypotheses have been proposed. The first one is the optophonic effect. A rapid local increase in temperature deriving from water absorption of photons produces a transient acoustic wave which can trigger depolarizing response of hair cells [7–9]. The second hypothesis is the photothermal activation of heat-sensitive ionic channels in the membrane of spiral ganglion cells, such as TRPV4 channels [10]. The third is that rapid local heating by laser can alter the electrical capacitance of the nerve membrane to evoke nerve excitability [11]. However, all the three mechanisms are related to photothermal effects [12, 13]. While, applying photothermal effects in the cochlea bears a potential risk of thermal damage. In future clinical applications, it is important to set safe parameters to have a good spatial selectivity and avoid excessive heating in the human cochlea.

In the human cochlea, spiral ganglion cells in the first turn of the coiled duct which accounts for high auditory frequency are located in the external part of the modiolus, while the ganglion cells that account for low frequency in the second and the third turn are located in the internal part of the modiolus. In the approach of photothermal stimulation, heat diffusion from the laser irradiated zone to the modiolus may reduce the laser selectivity. Therefore, it is necessary to take a quantitative overview of the temperature variations in the spatial domain and assess the effects of the heat diffusion on the laser selectivity. However, there is a lack of studies to date into directly assessing laser heating in humans due to the large body of evidence required to demonstrate safety and efficacy.

Fortunately, the theoretical modeling offers an appropriate method to analyze the temperature variation in the spatial and temporal domain [14–16]. For instance, in the model developed by Thompson et al. [14, 15] the cochlea is represented by a three-layer system: perilymph, nerve tissue, and a bone layer between nerve and perilymph. A range of fiber numerical apertures and light wavelengths were compared regarding stimulation of nerves in the cochlea. Zhang et al. [16] simplified the spiraled cochlea to a rotational symmetrical structure, and simulated infrared laser heating of the human cochlea for a range of laser pulse energy and repetition rates. These studies confirmed that laser heating in the cochlea can be controlled by properly adjusting laser parameters.

Until now, most of the research in this field focused the attention on animals, which has produced a number of valuable results. The research on animals is essential to explore the mechanism of laser stimulation and can be a useful reference for future investigations of laser application in the human cochlea. From the perspective of laser safety, it is interesting to examine if studies on animals can offer a clue to the safe laser parameters in the human cochlea. Therefore it is useful to make a contrastive analysis of laser heating in the cochlea of animals and human beings.

Considering that guinea pigs have been widely used in experiments, this work also chose the guinea pig as the contrastive object. The temperature variation in the guinea pig and human cochlea was simulated with a 3D model solved with the finite element method. In particular, the difference of laser heating in the human and guinea pig cochlea was analyzed.

## Methods

**Fig. 1:**
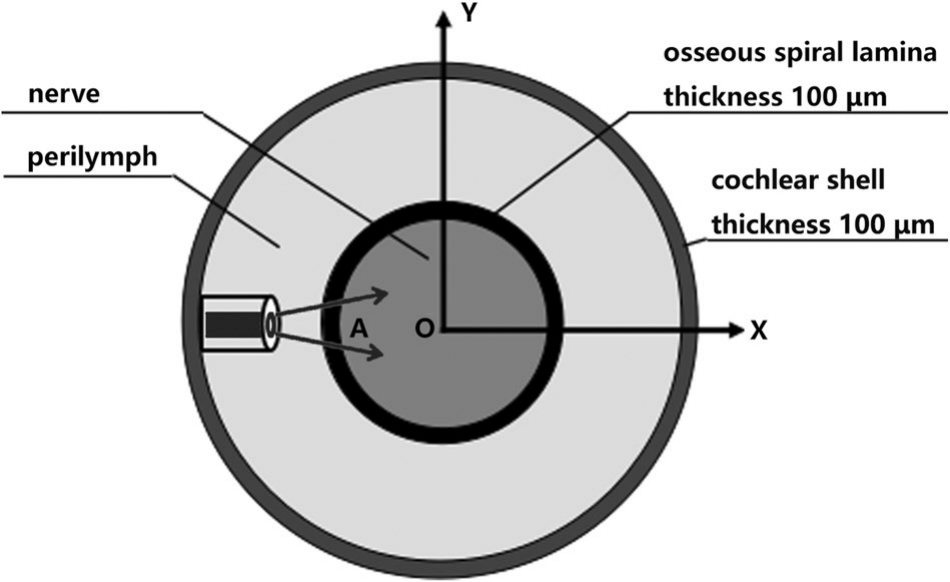
Illustration of the four-layer cylindrical model for simulations in XY plane. Laser light is delivered by an optical fiber which is placed 200 μm far from the osseous spiral lamina. Light passes through the perylimph and the osseous spiral lamina, and reaches the nerve layer. Characters* A* and* O* mark two representative sites where the temperature rise vs, time is presented below. The diameter of the cochlear shell is 6 mm for humans and 3 mm for guinea pigs. The diameter of the cochlear nerve core is 2 mm for humans and 0.8 mm for guinea pigs

**Fig. 2:**
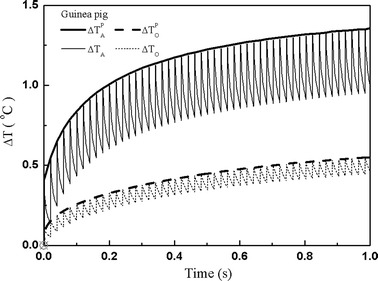
Temperature change (ΔT_A_, ΔT_O_) at two typical sites (A and O as marked in Fig. 1) in the cochlea of the guinea pig (laser pulse energy E = 45 μJ with pulse rates of 50 Hz). Site A represents the nerve layer, 100 μm underneath the osseous spiral lamina, and site O represents the center of the modiolus. The *bold solid line* and *dash line stand* for the peak temperature rise at site A and site O respectively

In this study, we extended the three-layer model developed by Thompson et al. to a four-layer cylindrical model [14, 15]. Figure 1 illustrates the cross-section of the model in the XY plane. It consists of perilymph, nerve tissue, a bone layer between nerve and perilymph, and a bony shell which encloses the cochlea. In this model, the same approximations were made as those in our previous work [16]. A brief outline of the approximations is given as follows:

(1) The scala vestibuli, scala tympani and scala media are modeled as one chamber, marked by perylimph as shown in Fig. 1; (2) the two cochleae share the same optical and thermal properties; (3) the loss of laser heating at the interface between the cochlea and the middle ear is overlooked; (4) in the case of laser stimulation, an acceptable temperature rise is usually only a few degree which enables the stimulation to be achieved without any tissue damage [17]. Thus, the thermal radiation is negligible as it depends on *T*^4^ (Stefan-Boltzmann law) [18]; (5) the thermal convection in tissues is also negligible since the perfusivity of most tissues is low [19]. As a consequence, attention is mainly focused on heat conduction in this simulation. The time-dependent heat equation is presented in the following equation [16, 18],

1 $$c\rho \frac{\partial T}{\partial t} - \nabla \times ( - k\nabla T) = Q(\vec{r},t)$$

where c is the heat capacity at constant pressure (J/kg·k), ρ is the density (kg/m^3^), and k is the thermal conductivity (W/m·k). In Eq. 1, Q represents the laser power density (W/m^3^) in the cochlea for single site stimulation, written as follows,

2 $$Q(\vec{r},t) = Q_{0} \cdot e^{{ - \alpha \left[ {\vec{k} \bullet (\vec{r} - \vec{r}_{0} )} \right]}} \cdot e^{{ - \frac{{\left| {\vec{k} \times (\vec{r} - \vec{r}_{0} )} \right|^{2} }}{{(\omega + N_{a} \cdot \vec{k} \bullet (\vec{r} - \vec{r}_{0} ))^{2} }} \cdot \exp ( - \mu_{s} \cdot \vec{k} \bullet (\vec{r} - \vec{r}_{0} ))}} \cdot P(t)$$

where *Q*_0_ is the power density at the outlet surface of the optical fiber which has a diameter of ω and a numerical aperture of *N*_*a*_, α is the light absorption coefficient, $\vec{k}$ represents the unit vector of the fiber direction, $\vec{r}_{0}$ is the coordinate of the fiber output surface, *μ*_*s*_ represents the scattering coefficients of tissues, and P(t) is a time-dependent dimensionless function representing the laser pulse-trains. The thermo-physical and optical properties of the cochlear tissues in the modeling are the same as those given in the literature, and listed in Table 1 [14–16, 20, 21].

**Table 1:** Physical properties of cochlear tissues

Tissues	Heat capacity (J/kg/ °C)	Density (kg/m^3^)	Heat conductivity (W/m/ °C/)	Absorption coefficient (mm^−1^)	Scattering coefficient (mm^−1^)
Modiolus	3.60 × 10^3^	1.05 × 10^3^	0.51	4.0	0.45
Perilymph	4.18 × 10^3^	1.00 × 10^3^	0.58	8.0	0.0
Bone	1.30 × 10^3^	1.90 × 10^3^	0.32	0.53	3.6

The modeling of laser heating in the cochlea of the guinea pig and human beings prominently differs in their boundary conditions. The human cochlea is located in the skull which is considered as a heat reservoir with a constant body temperature. On the contrary, the guinea pig cochlea is located in the temporal bone filled with air. Thus, we assume that the guinea pig cochlea loses heat mainly via air convection. Another difference between the two cochleae is the cochlear size. The diameter of the cochlear shell is 6 mm for humans and 3 mm for guinea pigs. The diameter of the cochlear nerve core is 2 mm for humans and 0.8 mm for guinea pigs.

The laser wavelength is set to be 1900 nm, and the laser pulse energy and pulse length are kept at 45 μJ and 100 μs respectively, as these laser parameters have been generally utilized in a number of studies [4, 15, 22–24].

For the given laser parameters, the model (Eq. 1) was solved by means of the finite element method with the COMSOL Script 1.3. The mesh elements are set in a tetrahedron shape with different sizes which are set to be small in the laser irradiated zones and slowly increased as the region moves far away from the laser stimulated sites. In total, the 3D model is divided into approximately 40,000 elements and 8000 mesh points.

When laser pulses are applied to stimulate the cochlea, the spiral ganglion cells absorb photons and become hot. Three typical sites in the human cochlea and two sites in the guinea pig cochlea were chosen, aiming to show how temperature changes if giving laser heating. One site, called A, represents the nerve layer 100 μm underneath the osseous spiral lamina, one site, called O, represents the center of the modiolus, as illustrated in Fig. 1. And the last site, called O^h^, is located between site A and O in the nerve layer of the human cochlea and has the same distance from the fiber as site O is in the guinea pig. To present a simpler illustration, the site O^h^ is not displayed in Fig. 1.

## Results and discussions

We first calculated the temperature change at site A and O in the cochlea of the guinea pig. As shown in Fig. 2, when the laser starts to heat, the temperature of the auditory tissues increases immediately. When irradiated by laser pulses at a repetition rate of 50 Hz, the spiral ganglion cells at site A experience a peak temperature rise of about 1.4 °C in merely one second, and the nerves in the center of the modiolus (site O) experience a rise of 0.55 °C. These results are in agreement with the estimations given by Zhang et al. and Izzo et al. [16, 17]. In addition, an oscillation of the temperature rise at both sites is observed, which results from photon absorption directly by the auditory tissues. After one pulse heating, the tissues cool down via heat diffusion. When the sites are heated by the following laser pulse, their temperatures increase rapidly before decrease again. Such processes are repeated with the laser repetition rate leading to the oscillation of the temperature change. Because site A is located closer to the fiber, the photon density is higher than that at site O, which causes greater oscillation at site A.

**Fig. 3:**
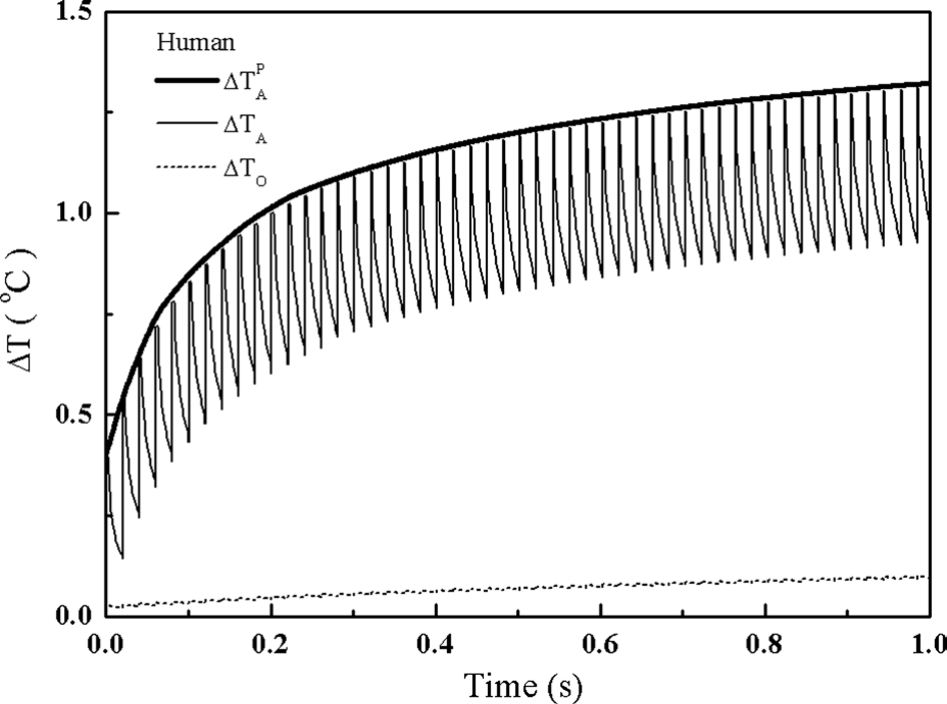
Temperatures changes of the human cochlea at site A and site O. The *bold solid line* represents the rise of peak temperature at site A. (The laser parameters are the same as those in Fig. 2)

Laser heating in the human cochlea follows a similar pattern to that in the guinea pig cochlea. As shown in Fig. 3, the auditory tissue at site A undergoes an initial sharp rise in temperature. Then, the temperature climbs slowly as more laser pulses are applied. However, comparing the laser heating in the cochleae of the guinea pig and human beings, two differences are found. The first concerns the peak temperature. The results show that the guinea pig cochlea gets hotter than the human cochlea in the same given time for laser heating. As shown in Figs. 2 and 3, the temperature at site A in the guinea pig cochlea increases about 1.4 °C, while it increases about 1.35 °C at the corresponding site of the human cochlea. However, the difference of laser heating is obvious at the center of the modiolus. The results show that the peak temperature at site O increases by 0.55 °C in the guinea pig, while it is only 0.13 °C in the human cochlea.

The second difference is the fluctuation of temperature rise at site O. There is a greater fluctuation of temperature rise in the guinea pig cochlea, compared with that in the human cochlea as reported in our previous work [16]. In the cochlea of the guinea pig, the nerve tissue at site O is located in the laser beam and directly absorbs heat from laser pulse trains. Therefore its temperature oscillates following the laser repetition rate. However, the nerve tissue at site O in the human cochlea is far from the laser irradiated zone, it receives heat via heat diffusion from the laser heated zone, which is a slow process. As a consequence, no obvious fluctuation in the temperature is observed.

**Fig. 4:**
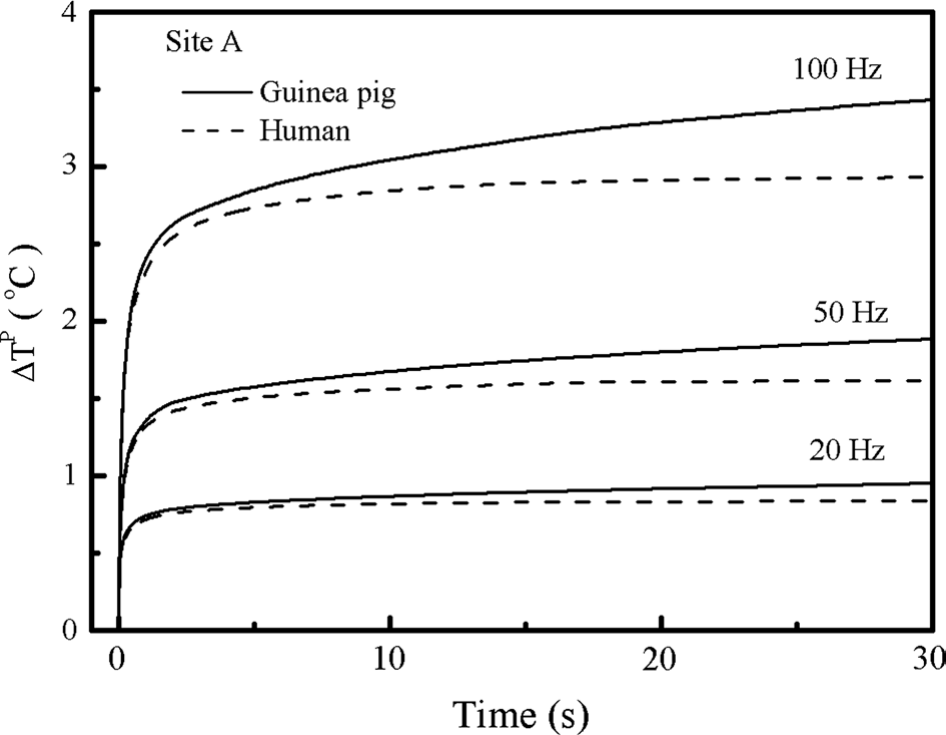
Peak temperature changes of the two cochleae at site A within 30 s. The same laser parameters utilized here as that mentioned* above*

In order to have a clear picture of the difference between the effects of laser heating in the two cochleae, we calculated and compared the peak temperature rise, *ΔT*^*P*^, at site A with respect to the heating time, when stimulated by laser pulse-trains for 30 s with the given pulse energy of 45 μJ. As presented in Fig. 4, the tissues at site A get hot immediately upon laser stimulation. After a few seconds of heating, the cochleae reach a quasi-steady thermal state in which the peak temperatures increase slowly for further successive pulses heating. For the same laser parameters, the guinea pig cochlea is a little bit hotter than the human cochlea, but the difference of temperature rise becomes more notable as the heating continues until the steady thermal state is reached. Our calculations found that it takes about 40 s for the human cochlea and 120 s for the guinea pig cochlea to reach a steady thermal state. In addition, the results show that the higher the repetition rates of laser heating is, the hotter both cochleae would become.

**Fig. 5:**
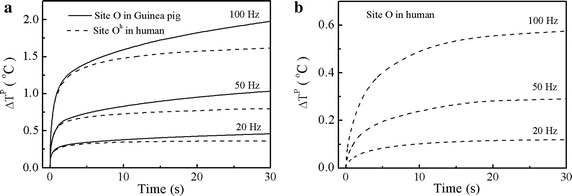
Peak temperature changes of the two cochleae at site O and O^h^ within 30 s. The laser parameters mentioned above are also applied as here

The trend of laser heating at site O and O^h^ is similar to that at site A, as presented in Fig. 5. It shows that, as more laser pulses are applied, heat accumulation in both cochleae becomes notable. Similar to the difference of laser heating at site A between the two cochleae, the laser heating at site O in the guinea pig cochlea is little bit higher than that at site O^h^ in the human cochlea since they are the same distance from the optical fiber. However, the temperature rise at site O in the human cochlea is obviously less than that in the guinea pig. As shown in Fig. 5, after 30 s of laser heating at the repetition rate of 100 Hz, the peak temperature rise at site O is about 1.90 °C in the guinea pig cochlea, but it is only 0.55 °C at site O in the human cochlea. In brief, our calculations show that, for the same laser parameters, the guinea pig cochlea gets hotter than the human cochlea.

The differences between the laser heating in the guinea pig cochlea and the human cochlea mainly result from two things. One is the effect of the different thermal boundaries of the two cochleae. The human cochlea is located in the temporal bone where there are plenty of blood capillaries, making its temperature more or less the same as body temperature. Thus, the temperature at the boundary of the human cochlea is set to be constant. However, there is an air gap between the guinea pig cochlea and the closed bulla. Through the air gap, there are two ways transferring heat from the cochlea to the bulla: radiation and convection. By assuming a temperature difference of 1 °C across the gap, an estimation of the power radiated from the guinea pig cochlea can be obtained as being 7 W/m^2^, and the power loss via air convection is calculated as being about 20 W/m^2^ by applying Newton’s law of cooling [25]. However, if the air gap was filled with bone, the heat transfer would be dominated by heat conduction. For the same temperature difference of 1 °C, the power loss via heat conduction can be estimated as being 300 W/m^2^ following the Fourier’s law of heat conduction [18]. Therefore, in a situation of low temperature, heat flows from the cochlea of the guinea pig to its bulla is a slower process compared to the heat conduction to the temporal bone in the human cochlea. The second reason for the difference induced is the effect of the cochlear size. Because the guinea pig cochlea looks smaller than the human cochlea, with the same laser energy, the guinea pig cochlea can justifiably be hotter than the human cochlea.

**Fig. 6:**
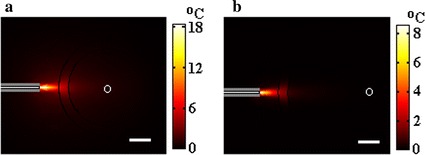
Distribution of the temperature rise in the cochlea of guinea pig and human after heating by a laser pulse-train with laser pulse energy of 45 μJ and repetition rate of 50 Hz for 120 s. **a** Temperature rise in the guinea pig cochlea, showing a maximal temperature of 18 °C in the perilymph. **b** Temperature rise in the human cochlea, presenting a maximal temperature rise of 8.5 °C in the perilymph

Although the laser heating in the guinea pig cochlea is higher than that in the human cochlea as presented in Figs. 2, 3, 4 and 5, our calculations found that the localization of laser stimulation in both cochleae is still high. In Fig. 6, we present the distribution of temperature rise in a XY plane after 120 s of pulse-train heating in both cochleae. In the simulation, the laser pulse energy is 45 μJ and the laser pulse repetition rate is 50 Hz. A good selectivity is obtained in both cochleae. However, current cochlear implants use electrical stimulation rates about 900 Hz [26]. In this context, a higher stimulation rates will possibly be applied in further studies on optical cochlear implants, indicating that greater heat may be produced. Therefore, more efforts are required for further investigations of the laser heating in the cochlea.

In summary, as reported in our previous work [13], the laser-affected zone is larger than the laser illumination due to heat diffusion, but our results show that: (1) The temperature rise in the modiolus is less than that in the laser-targeted ganglion cells; (2) Laser heating in the cochlea of the guinea pig is higher than that in the human cochlea; (3) the laser heating in both cochleae is confined mainly to the laser target region. Our results indicate that the cochlea of guinea pig could be an acceptable model for the sake of laser safety. Therefore, future experimental studies in the guinea pig cochlea could help to set proper laser parameters for laser stimulation of the human cochlea.

Due to the lack of information detailing physical properties of the cochlear tissues, including heat conductivity, heat capacity and optical absorption coefficient, in this work, the physical properties were acquired by analyzing the data of similar tissues described in literature. In addition, the spiraled cochlea was simplified as a four-layer cylinder. In future studies, the modeling of laser heating can be improved with more precise setting of the physical parameters and more careful consideration of the spiraled cochlear structure, as well as much attention on the influence of thermal convection via blood flow and other fluids flow on laser heating.

## Conclusions

Infrared laser heating in the cochlea of the guinea pig and human was investigated by applying a 3D four-layer cylindrical model. Two stages in the laser heating were observed. In the first stage, the two cochleae display a sharp increase in temperature. In the next stage, the cochleae enter a quasi-steady stage in which the peak temperature rise changes slowly as more laser pulses are applied. Moreover, the temperature in the cochlea of guinea pig is higher than that in the human cochlea. It is a result of the difference in the cochlear size and the boundary thermal conditions for the cochlea of the guinea pig and humans. This study indicates that, from the perspective of laser safety, future experimental studies in the guinea pig cochlea could help set proper laser parameters for laser stimulation of the human cochlea.
